# Oxidative damage from repeated tissue isolation for subculturing causes degeneration in *Volvariella volvacea*

**DOI:** 10.3389/fmicb.2023.1210496

**Published:** 2023-07-20

**Authors:** Fengyun Zhao, Qiaoli Wang, XueMing An, Qiangfei Tan, Jianmin Yun, Yubin Zhang

**Affiliations:** ^1^College of Food Science and Engineering, Gansu Agricultural University, Lanzhou, China; ^2^Kangle County Special Agricultural Development Center, Linxia, Gansu, China; ^3^Lanzhou Institute of Biological Products Limited Liability Company, Lanzhou, Gansu, China

**Keywords:** *Volvariella volvacea*, tissue isolation, oxidative damage, mitochondrial, succession degeneration, senescence

## Abstract

The fungal fruiting body is the organized mycelium. Tissue isolation and mycelium succession are common methods of fungal species purification and rejuvenation in the production of edible mushrooms. However, repeated succession increases strain degeneration. In this study, we examined the effect of repeated tissue isolation from *Volvariella volvacea* fruitbodies on the occurrence of degeneration. The results showed that less than four times in succession improved production capacity, however, after 12 successions, the traits indicating strain degeneration were apparent. For instance, the density of aerophytic hyphae, hyphal growth rate and hyphal biomass were gradually reduced, while the hyphae branching was increased. Also, other degenerative traits such as prolonged production cycles and decreased biological efficiency became evident. In particular, after 19 successions, the strain degeneration became so severe no fruiting bodies were produces anymore. Meanwhile, with the increase in successions, the antioxidant enzyme activity decreased, reactive oxygen species (ROS) increased, the number of nuclei decreased, and the mitochondrial membrane potential decreased along with morphological changes in the mitochondria. This study showed that repeated tissue isolation increased oxidative damage in the succession strain due to the accumulation of ROS, causing cellular senescence, in turn, degeneration in *V. volvacea* strain.

## 1. Introduction

*Volvariella volvacea*, a grass-rotting fungus, is one of the most widely cultivated edible mushrooms in the tropics and subtropics (Chonticha et al., 2021). It is also known as the Chinese mushroom due to its cultivation originated in China. It is a nutritious, tasty fungus with good health benefits and an important export-earning mushroom in China (Xu X. et al., 2019). In industrial production, methods of asexual reproduction such as mycelium subculture and the generation of tissue cultures from fruiting bodies are often used to purify and rejuvenate *V. volvacea* strains. However, repeated tissue preparation can lead to strain degeneration characterized by decline in viability and reduced nutrient content (Chen et al., 2019).

Similar to other edible mushrooms, strain degeneration is an important limiting factor in the development of *V. volvacea*. Degenerate strains suffer from slow mycelial growth, delayed mushroom emergence, decreased yield, and weakened resistance, all of which significantly impact the industrialization of edible mushrooms (Li H. B. et al., 2020). Cryopreservation is an effective method to slow down the decline in strain quality by reducing mycelial growth and the occurrence of genetic mutations (Liu et al., 2018). However, *V. volvacea* is a high-temperature growing mushroom and therefore does not tolerate low-temperature storage. At 4°C, it dies by autolysis within 48 h (Zhao et al., 2018). Therefore, strain degeneration is a particularly serious problem in the case of *V. volvacea*. Strain degeneration in edible mushrooms has been associated with excessive accumulation of reactive oxygen species (ROS) (Pérez et al., 2021).

ROS is a general term for oxygen-containing substances with active chemical properties and strong oxidative ability (Mansoor et al., 2022). During the life activities of aerobic organisms, mitochondria and the plasma membrane-associated electron transport system generate various ROS. Under normal conditions, ROS are detoxified by various antioxidant defense mechanisms (Halliwell, 2006). However, excessive ROS production can lead to oxidative damage to many cellular components, such as lipids, proteins, and DNA, triggering cell death (Dong et al., 2009; Holley et al., 2011). Mitochondria are energy-producing organelles in eukaryotic cells and play an important role in maintaining intracellular calcium homeostasis, signal transduction, and apoptosis (Zhu et al., 2019). Mitochondria are also the main site of ROS generation and accumulation under various stresses. An uncontrolled ROS accumulation causes mitochondrial dysfunction, resulting in cell death and then tissue damage (Kirkham and Barnes, 2013).

Fungi normally scavenge ROS through an oxidoreductase system (Chen et al., 2020). In *Cordyceps militaris*, increased glutathione peroxidase (GPX) activity was shown to participate in ROS scavenging, indicating that antioxidant genes can play an effective role in reversing fungal degeneration during successive cultures (Liu et al., 2018). The activities of antioxidant enzymes superoxide dismutase (SOD), catalase (CAT), glutathione reductase (GR) and GPX were found directly associated with ROS scavenging in *Morchella importuna*, affecting the mycelial growth and fruiting body development (Bai et al., 2021). This study examined the changes in production traits of *V. volvacea* degenerated strains after multiple successions, in light of the change in ROS accumulation, antioxidant enzyme activity, mitochondrial morphology, and other indicators. Our results suggested that strain degeneration of *V. volvacea* was associated with and likely caused by ROS accumulation, which provided basis for studying the degradation mechanism of *V. volvacea* and other fungi.

## 2. Materials and methods

### 2.1. Strains and media

The original strain (T0), a commercially cultivated strain V844, was conserved in the College of Food Science and Engineering, Gansu Agricultural University, China.

Succession strains were obtained by tissue isolation as follows: T0 was cultivated, egg-shaped fruiting body were obtained, the fruiting body were cut open, and a small piece was cut at the stalk-cap junction to generate the first generation tissue culture, named T1. The same method was followed 19 times to obtain total of 19 generations named T1–T19 (Supplementary Figure S1), with three replicates of each strain. All strains were stored in liquid paraffin at 20°C.

Media for strain cultivation were as follows. Potato dextrose broth (PDB) medium: 200 g fresh potato, 20 g glucose, 1.0 g KH_2_PO_4_, 1.0 g MgSO_4_-7H_2_O, in 1000 mL distilled water. Potato dextrose agar (PDA) medium: 20% agar in PDB. Spawn substrate/cultivation medium (w/w): 88% cotton seed hulls, 10% bran, 1% gypsum, 1% lime, and 65% water content (pH 8–9). Cultivation substrate (w/w): 97% waste cotton, 3% lime, and 70% water content (pH 9–10).

### 2.2. Observation of colony and mycelium

The colony and mycelium were examined as described by Fu et al. (2015). All strains were inoculated onto PDA medium and the colony morphology of the succeeding strains was observed and photographed after 3 days of incubation at 30°C.

### 2.3. Measurement of mycelial growth rate

The mycelial growth rate was determined according to Chung et al. (2020). A mycelium block of 1 cm diameter was taken with a hole punch and inoculated in the center of a PDA plate. After incubation for 72 h at 30°C, the colony diameter was marked on the plate by the cross-marking method and the mycelial growth rate was calculated as follows:


$$\begin{array}{l} \mathrm{Mycelial growth rate}\;\left(\mathrm{mm}/h\right)=\\ \frac{\;\left(\mathrm{Colony diameter}-\mathrm{Inoculated mycelial block diameter}\right)\;\mathrm{mm}}{72\;h} \end{array}$$

### 2.4. Determination of mycelium biomass

Mycelium biomass was determined by referring to the method of Li et al. (2020). The cellophane was cut into a size slightly smaller than the petri dish, and after sterilization, the sterile cellophane was covered on the surface of the solidified PDA plate. The respective strains were inoculated with 1 piece of mycelium (6 mm diameter punch) on PDA plate at 30°C for 3 days. The mycelium was then gently scraped off, weighed, and the data were recorded.

### 2.5. Determination of mycelial branching

Mycelial branching was determined as described by Guo et al. (2020). *V. volvacea* strains T0, T4, T8, T12, T16, and T19 that were preserved in liquid paraffin were taken out for uniform activation in triplicates. The mycelial blocks (1 cm diameter) of respective strains were inoculated in the center of a PDA plate, a clean sterile slide was inserted into the plate at an angle of 45°, and the plate was incubated at 30°C for 3 days. Afterward, the plate was opened, the slide was gently removed. The slide was placed under a light microscope for observation and photography, and the number of mycelial branches of each strain was counted in the same field of view.

### 2.6. Measurement of cell nuclei number

The cell nuclei number was estimated according to Gao et al. (2019). The mycelia of T0, T4, T8, T12, T16, and T19 strains were grown on PDA plates at 30°C for 3 days as described in Section 2.5. When the mycelium grew to occupy about 1/3rd of the inserted slide, 10 μL of DAPI (2-(4-amidinophenyl)-6-indolecarbamidine dihydrochloride) staining solution was poured onto the slide, the excess dye was removed with the help of a tissue paper. The DAPI-stained slide was observed under an inverted fluorescence microscope and the number of nuclei per 100 micron length of mycelia was counted.

### 2.7. Determination of cultivation characteristics

Cultivation characteristics were determined following the method of Liu et al. (2011) with slight modifications. T0–T19 strains were cultured uniformly on PDA plates for 5 days. Then, three mycelium blocks (10 mm diameter) were cut off and transferred to 500 mL seed medium in a culture flask for incubation at 30°C for 10 days. Later the culture was inoculated in plastic frames (40 cm × 20 cm × 10 cm) with 1 kg of cultivation medium. Three parallels were set for each strain. During the cultivation period, parameters such as the primordium formation time, fruiting body diameter, fruiting body number, production cycle, and biological efficiency of the respective strain were recorded.

Primordium formation time denoted the number of days required for the first primordium to sprout from inoculation to cultivation medium. Production cycle denoted the number of days required for the fruiting body to grow into the harvest period, i.e., the egg-shaped period, after inoculation to the cultivation medium. The number of fruiting bodies denoted the number of sub-entities in the egg-shaped period in the respective plastic frame during the harvest period. The diameter of the fruiting body was calculated as the average value from 5 randomly selected egg-shaped fruiting bodies using a vernier caliper. Biological efficiency was calculated as follows:


$$\mathrm{Biological efficiency}\;\left(\%\right)=\frac{\;\left(\mathrm{Fresh fruiting body yield}\right)}{\left(\mathrm{Quantity of}\;\mathrm{dry}\;\mathrm{substrate used}\right)}\times 100\%$$

### 2.8. Nitroblue tetrazolium chloride staining

Nitroblue tetrazolium chloride (NBT) staining was performed as described by Parkhey et al. (2012). T0, T4, T12, and T19 strains were uniformly activated. A mycelial block (1 cm in diameter) from the respective strain was attached to the PDA medium with a punch and a slide was inserted into the medium. The strain was incubated at 30°C for 3 days. When the *V. volvacea* mycelium grew to occupy 1/3rd of the slide, staining was performed with 0.3 mmol/L NBT working solution at room temperature for 20 min. 30% glycerol was added onto the coverslip before observation under a light microscope.

### 2.9. Determination of ROS content and antioxidant enzymes activities

T0-T19 strains were inoculated in the PDB medium for 3 days and the mycelium was collected. The intracellular levels of hydrogen peroxide (H_2_O_2_), superoxide anion (O_2_^−^) and antioxidant enzymes (SOD, CAT, GPX, and GR) were estimated using commercial kits (Beijing Solaibao Technology Co., Ltd., Beijing, China) following the manufacturer’s instructions.

### 2.10. Estimation of antioxidative enzymes gene expression by quantitative real-time polymerase chain reaction

Quantitative real-time polymerase chain reaction (qRT-PCR) analysis of four antioxidant enzyme genes (*sod*, *cat*, *gpx*, and *gr*) was performed as described by Livak and Schmittgen (2001). The *V. volvacea* housekeeping gene, glyceraldehyde phosphate dehydrogenase (*gapdh*), was used as the internal reference gene. The respective primers were designed using Primer Premier 7.0 software (sequences were listed in Supplementary Table S1). *V. volvacea* V23 was used as a reference.^1^

T0–T19 strains were incubated in the PDB medium at 30°C for 4 days. Mycelium was collected and washed 2–3 times with sterile distilled water. The harvested mycelium was ground to a fine powder in liquid nitrogen. Total RNA from the mycelium powder was extracted using RNA simple Total RNA Kit [Tiangen Biochemical Technology (Beijing) Co., Ltd., Beijing, China]. PrimeScript^™^ RT Master Mix (TaKaRa, Shiga, Japan) was used to synthesize the first strand complementary DNA (cDNA), followed by RT-PCR amplification using a Real-Time PCR instrument (LightCycler Model 480, Roche, Germany). The relative gene expressions were calculated using the 2^−ΔΔCT^ method (Livak and Schmittgen, 2001).

### 2.11. Mitochondrial staining

Mitochondrial staining was performed as described by Xu et al. (2019). The activated T0, T4, T12, and T19 strains were removed on the ultra-clean bench with a sterilized 1 cm diameter punch. The corresponding mycelium block was inoculated in the center of a PDA plate, and a sterile slide (one per plate) was inserted obliquely into the medium at an angle of 45°. The plate was incubated at 30°C for 4 days. Afterward, the slide was rinsed 2–3 times with phosphate buffer saline (PBS) and then stained with Mito-Tracker Green for 20 min at room temperature. The excess stain was removed by washing with PBS 2–3 times and finally, the slide was observed under an inverted fluorescence microscope.

### 2.12. Measurement of mitochondrial membrane potential

Mitochondrial membrane potential was measured according to Moghadasi et al. (2019). The activated T0, T4, T12, and T19 strains were grown on a slide using the PDA plate as described in Section 2.5. When mycelium grew about 1/3rd of the slide, it was fixed in 3.7% paraformaldehyde, rinsed 2–3 times with PBS for 20 min, and then added with Mito-Tracker Red CMXRos dropwise for 20 min. The excess stain was washed 2–3 times with PBS (pH 7.5) and the slide was observed under an inverted fluorescence microscope.

### 2.13. Data processing

All experiments had three replicates and the measurement data were expressed as mean ± standard deviation (SD). Data were statistically processed using Microsoft Excel 2010, and one-way ANOVA significance of data was performed using SPSS19.0 (SPSS Inc., United States); Origin 9.0 was used for data plotting.

## 3. Results

### 3.1. Colony morphology and mycelial growth characteristics of *Volvariella volvacea* succeeding strains

**Figure 1:**
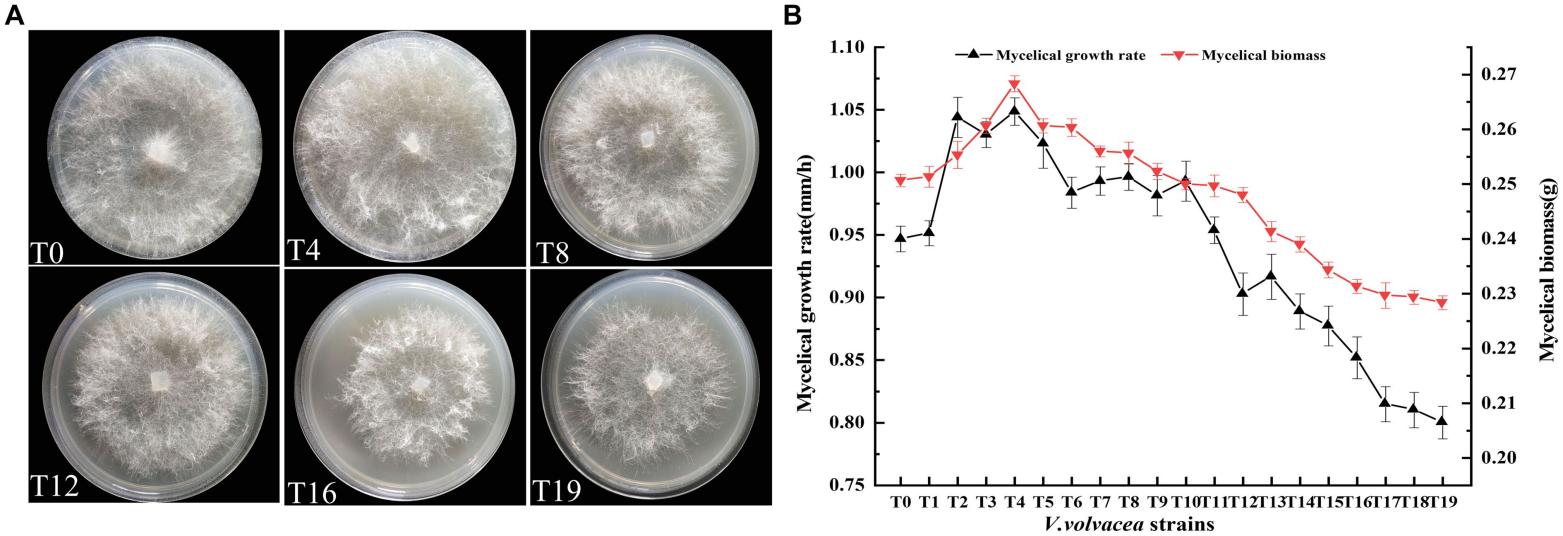
Physiological traits of *V. volvacea* mycelium from succeeding strains. **(A)** Colony morphology. **(B)** Changes in mycelial growth rate and biomass.

Total of 19 succession strains (T1–T19) of *V. volvacea* were obtained by tissue isolation. The strain T19 was obtained after 18 successions. We found that T19 failed to produce fruiting body. T0–T19 were incubated uniformly on corresponding PDA plates, and the colony morphology was observed and photographed after 3 days. The results were shown in Figure 1A. The change in aerial mycelial density was not obvious for T0 and T4. However, beginning from the T8 succession, with the increase in succession generations, the colony diameter gradually decreased, the aerial mycelium density became sparse.

With the increase in succession, both the mycelial growth rate and mycelial biomass first increased (up to T4) and then decreased (Figure 1B). There was an obvious positive correlation between mycelial growth rate and mycelial biomass. The mycelial growth rate began to decrease after peaking at T4 and further accelerated after T12. The mycelial growth rate of T19 was 14.7% lower than that of T0. Similarly, the mycelial biomass peaked at T4 and then decreased; the mycelial biomass of T19 was 8.9% lower than that of T0.

### 3.2. Changes in the numbers of mycelial branching and nuclei in *Volvariella volvacea* succeeding strains

**Figure 2:**
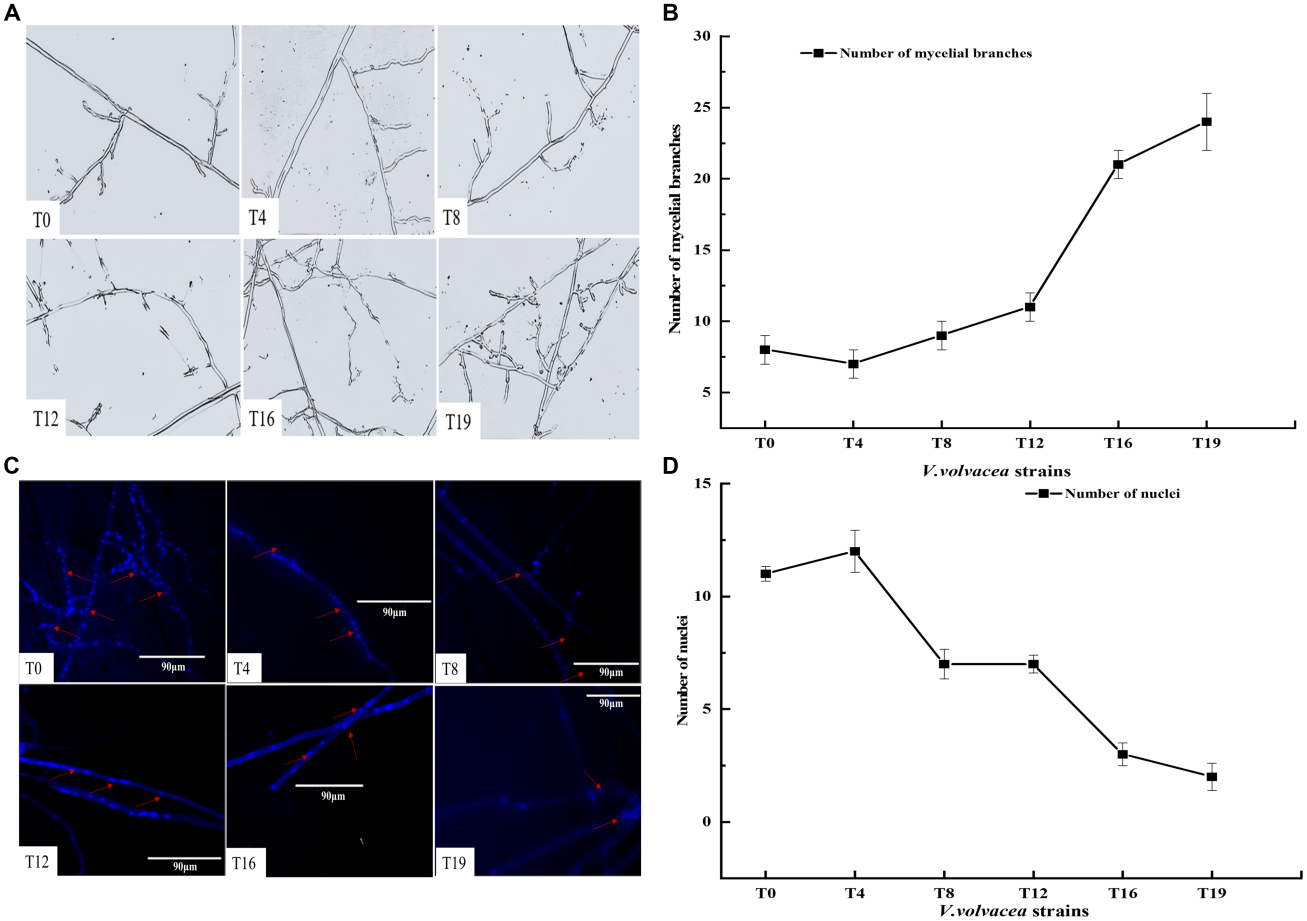
Changes in the numbers of mycelial branching and nuclei in *V. volvacea* succeeding strains. **(A)** Status of mycelium branching. **(B)** Change in the number of mycelial branching. **(C)** Nuclear staining. **(D)** Change in the number of nuclei.

Microscopic observations of the mycelium from succeeding strains were shown in Figure 2A. At the same magnification, the mycelium of T0 and T4 strains showed the least branches, however, the mycelium branching began to increase from the T12 generation. The T19 strain exhibited the highest branching of mycelium and the mycelium intertwined with each other into a network (Figure 2B).

The number of nuclei in mycelium was estimated by fluorescence microscopy. In general, the number of nuclei first increased and then decreased with the increase in succession generations (Figure 2C). The number of nuclei was the highest at T4 and the lowest at T19. The number of nuclei was smaller in T8, T12, T16, and T19 than in T0 (Figure 2D).

### 3.3. Changes in the production traits of *Volvariella volvacea* succeeding strains

**Figure 3:**
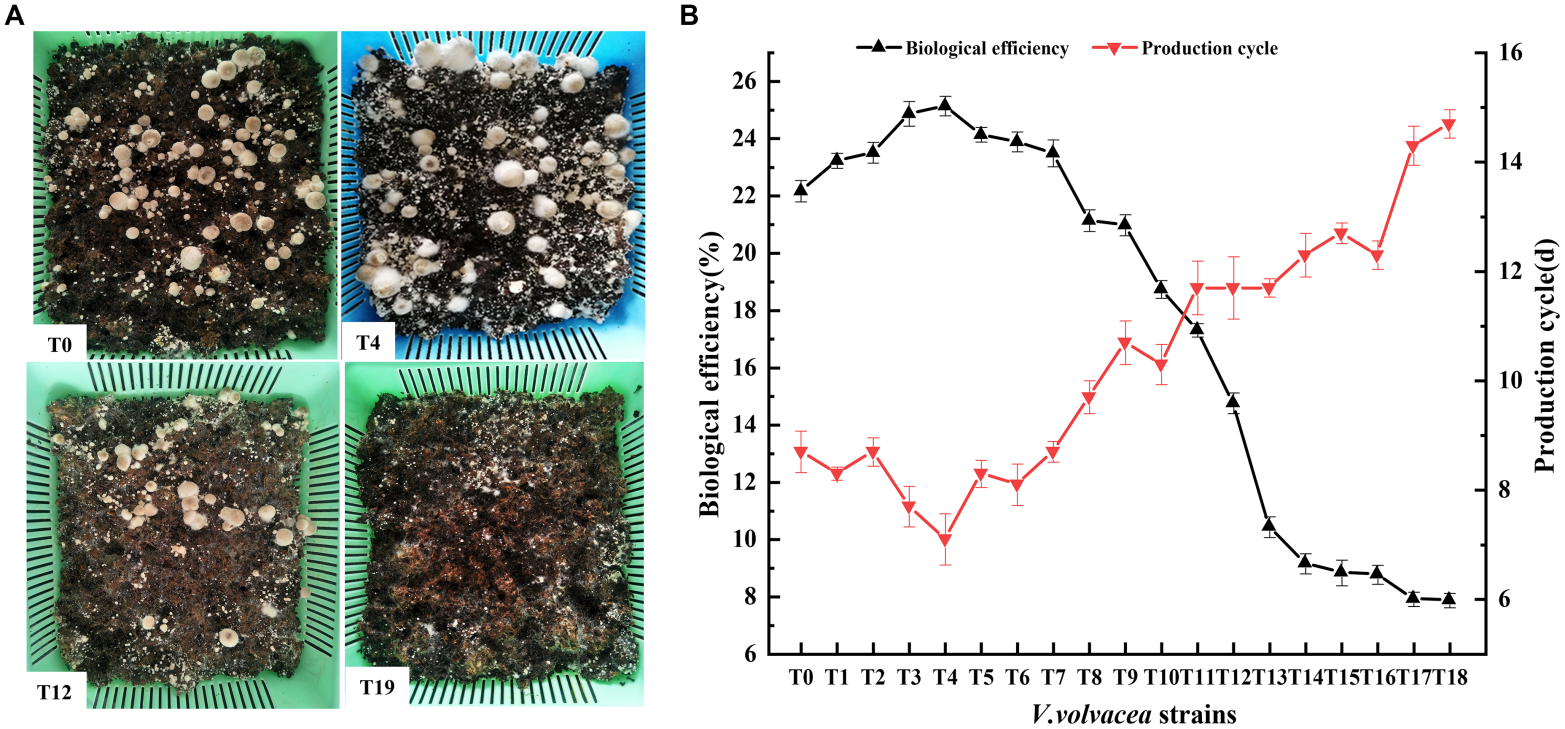
Production traits of *V. volvacea* succeeding strains. **(A)** Cultivation test. **(B)** Production cycle and change in biological efficiency.

We conducted uniform cultivation of *V. volvacea* succeeding strains and recorded their production characteristics. T0–T18 could produce fruiting bodys, while T19 failed to do so (Figure 3A). The production cycle gradually increased with the increasing number of passaged cultures. Compared with T0, the production cycle of T18 was prolonged by 6 days. Also, the increasing passage gradually decreased the biological efficiency of strains; T18 was at 7.87%, which was 64.50% lower than that of T0 (Figure 3B).

The number and diameter of the fruiting body first increased and then decreased with the increase in succeeding generations (Table 1). Meanwhile, the primordium formation time first decreased and then increased. The number and diameter of the fruiting body at the T4 generation were 40.78 ± 2.13 and 33.45 ± 1.15 mm respectively, which were 21.2% and 29.3% higher than those of T0; the number and diameter of the fruiting body at the T18 generation were 8.14 ± 1.65 and 19.03 ± 1.23 mm respectively, which were 75.8 and 26.5% lower than those of T0. The primordium formation time for T4 and T18 was 5.43 ± 0.83 (19.4% less than that of T0) and 9.63 ± 0.66 (43.3% more than that of T0) days, respectively (Table 1).

**Table 1:** Changes in the number of fruiting bodies, the diameter of the fruiting body, and primordium formation time of *V. volvacea* succeeding strains.

Strains	Number of fruiting bodies	Diameter of the fruiting body (mm)	Primordium formation time (d)
T0	33.66 ± 2.86	25.88 ± 0.41	6.74 ± 0.66
T1	31.78 ± 2.67	27.33 ± 1.22	6.53 ± 0.68
T2	32.33 ± 2.32	27.96 ± 0.56	6.06 ± 0.76
T3	34.34 ± 2.34	28.27 ± 0.63	6.87 ± 1.64
T4	40.78 ± 2.13	33.45 ± 1.15	5.43 ± 0.83
T5	35.13 ± 2.03	30.83 ± 0.67	5.17 ± 1.16
T6	31.00 ± 1.79	27.77 ± 1.03	6.53 ± 0.66
T7	27.33 ± 1.87	27.28 ± 1.18	6.59 ± 1.14
T8	25.43 ± 1.67	27.07 ± 1.36	7.43 ± 2.06
T9	23.63 ± 2.45	25.64 ± 0.56	7.60 ± 1.04
T10	20.63 ± 2.64	24.57 ± 1.23	7.43 ± 1.43
T11	20.53 ± 2.54	23.04 ± 1.52	7.93 ± 1.23
T12	15.74 ± 1.67	22.31 ± 1.54	8.13 ± 1.62
T13	13.75 ± 2.34	21.84 ± 1.52	8.60 ± 1.04
T14	10.37 ± 1.43	20.67 ± 1.23	8.77 ± 1.36
T15	8.07 ± 1.72	20.51 ± 2.47	9.33 ± 1.61
T16	7.73 ± 1.32	20.20 ± 2.14	9.33 ± 1.22
T17	8.56 ± 1.15	19.45 ± 1.34	9.53 ± 0.64
T18	8.14 ± 1.65	19.03 ± 1.23	9.63 ± 0.66

The values were the mean ± standard deviation (SD) of three strains.

### 3.4. Change in ROS content in *Volvariella volvacea* succeeding strains

**Figure 4:**
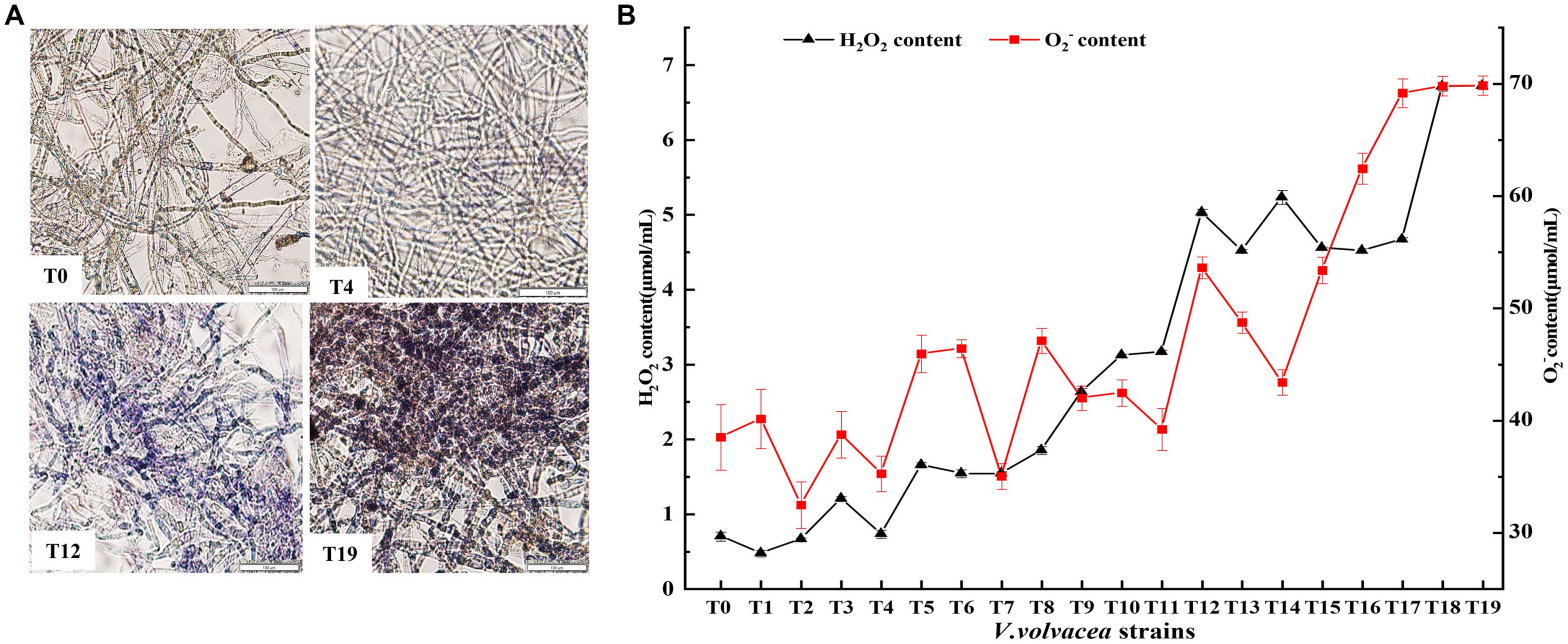
Change in ROS content in *V. volvacea* succeeding strains. **(A)** NBT staining. **(B)** Change in H_2_O_2_ and O_2_^−^ content.

We performed NBT staining to examine the change in ROS content in the mycelium of *V. volvacea* strains. The stained mycelia were shown in Figure 4A. Compared with later generations, the color of T0 and T4 mycelia was lighter but not so different from each other. As the number of generations increased, the mycelium color gradually deepened; the color of T12 mycelia was dark blue and the T19 mycelium-stained indigo. It indicated that the ROS content in the T12 and T19 strains was significantly higher than that in T0 and T4 strains.

The contents of O_2_^−^ and H_2_O_2_, the two main components of ROS, were measured (Figure 4B). The O_2_^−^ content showed slow increase in fluctuation until the T12 generation, and then rapid increase from T12 to T19; the O_2_^−^ content increased by 50.1% in the T19 strain compared with the T0 strain. The H_2_O_2_ content increased rapidly with the increasing generation; the H_2_O_2_ content was 8.6 times higher in T19 than in T0.

### 3.5. Change in antioxidant enzyme activities of *Volvariella volvacea* succeeding strains

**Figure 5:**
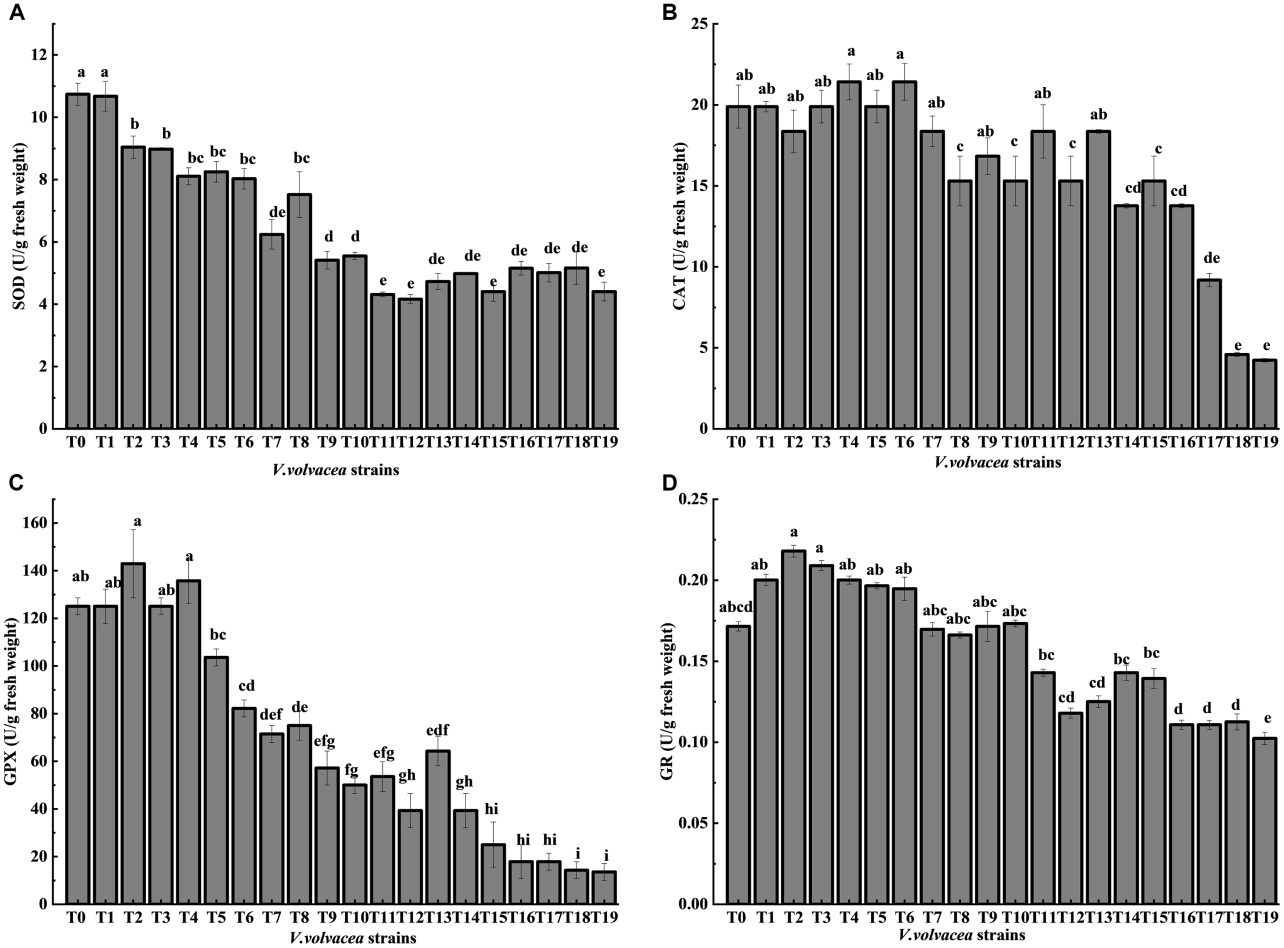
Change in antioxidant enzyme activities of *V. volvacea* succeeding strains. Enzyme activities of **(A)** SOD, **(B)** CAT, **(C)** GPX, and **(D)** GR.

The enzyme activities of ROS-scavenging antioxidant enzymes were measured in *V. volvacea* succeeding strains (Figures 5A–D). Both GPX and GR activities showed first increasing trend and then decreasing, while the SOD activity decreased rapidly with the increase in succession number until T8 and then became stable. The CAT activity remained relatively stable until T13. The differences were not significant for GPX activity until T4 and for GR activity until T10, which then decreased rapidly with the increasing generations. Compared with T0, at T19, SOD, CAT, GPX, and GR activities decreased by 59.0%, 77.1%, 88.6%, and 40.3%, respectively.

### 3.6. Relative expression of antioxidant enzyme genes in *Volvariella volvacea* succeeding strains

**Figure 6:**
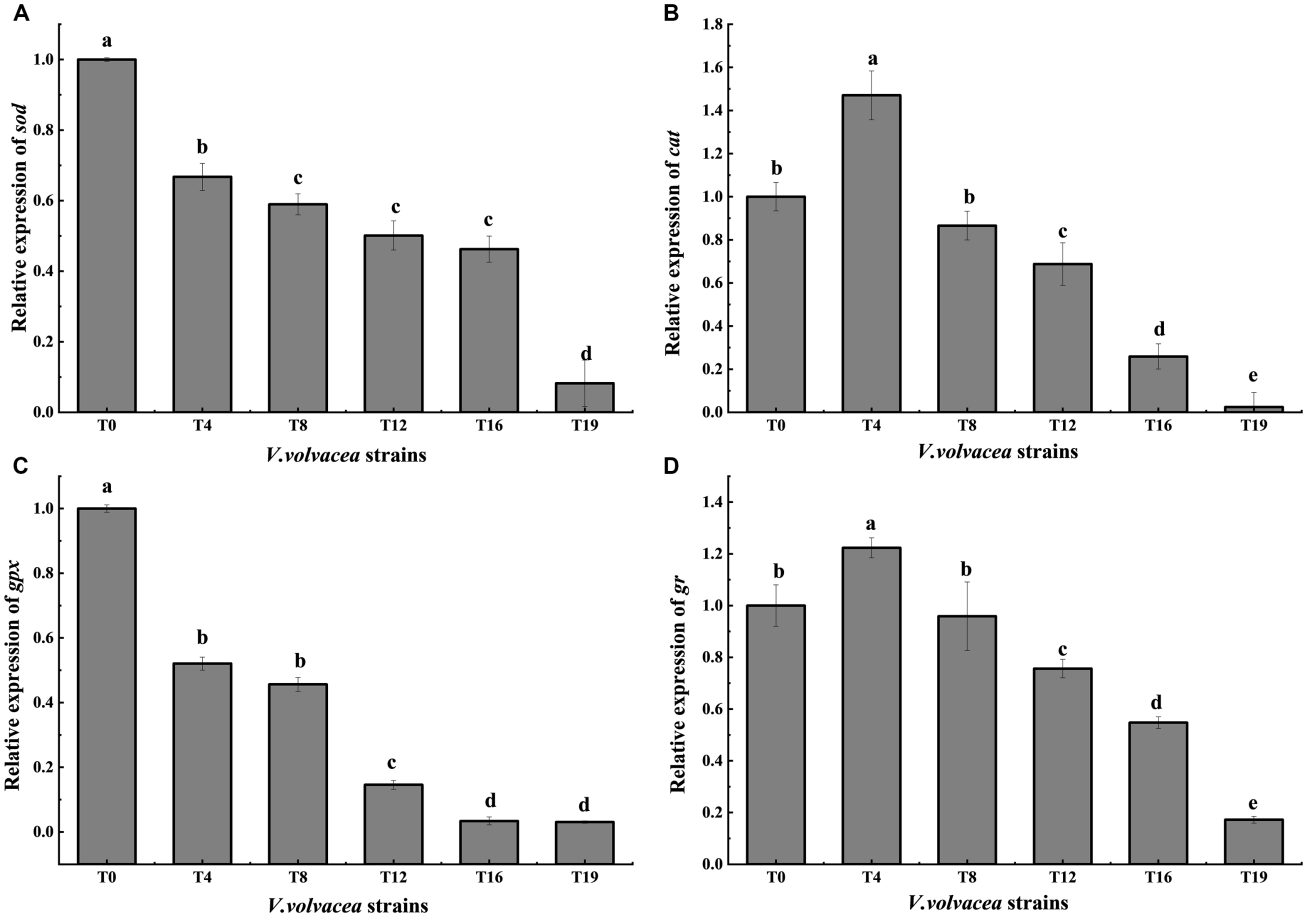
Relative expression of antioxidant enzyme genes in *V. volvacea* succeeding strains. Relative gene expression of **(A)** *sod,* **(B)** *cat* **(C)** *gpx,* and **(D)** *gr*.

The relative gene expression levels of *sod*, *cat*, *gpx*, and *gr* were analyzed by qRT-PCR using T0 as the control group strain and T4, T8, T12, T16, and T19 as the experimental group strains (Figures 6A–D). With the increasing generations, the expression of gr and cat first increased and then decreased (Figures 6B,D), meanwhile, the expression sod and gpx showed a decreasing trend (Figures 6A,C). All four genes were expressed at the lowest level in T19. Compared with T0, the expression levels of *sod*, *cat*, *gpx* and *gr* decreased by 91.78%, 97.53%, 96.94% and 82.76%, respectively, in T19. These results were not completely consistent with the results of enzyme activity assays, which could be attributed to specific translational regulation of the respective genes.

### 3.7. Mitochondrial changes in *Volvariella volvacea* succeeding strains

**Figure 7:**
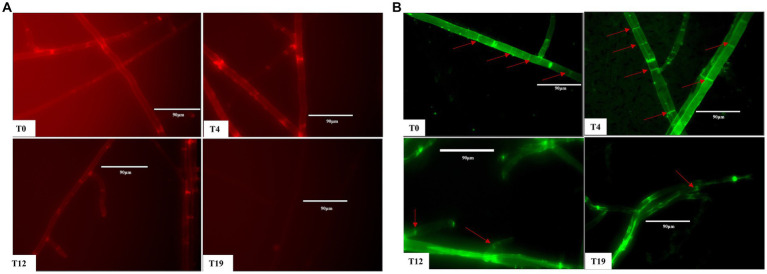
Mitochondrial changes in *V. volvacea* succeeding strains. **(A)** Mitochondrial membrane potential. **(B)** Mitochondrial morphology.

Staining tests for estimation of mitochondrial membrane potential and mitochondrial morphology were performed on T0, T4, T12, and T19 mycelia. After staining with mitochondrial membrane potential-dependent fluorescent dye probes, both T0 and T4 mycelia showed bright red fluorescence, the red fluorescence of T12 mycelium was significantly weaker, while T19 mycelium showed no obvious red fluorescence (Figure 7A). These results demonstrated that the increase in oxidative stress reduced the mitochondrial membrane potential in *V. volvacea* succeeding strains.

Accordingly, for mitochondrial morphology observation, we used the specific fluorescent dye Mito-Tracker Green to label the mitochondria. The staining results showed obvious bright spots in T0 and T4 mycelia, while the numbers of such spots were reduced in T12 mycelium and were almost absent in T19 mycelium (Figure 7B). These results indicated that mycelium mitochondria shape changed significantly with the increase in successions of *V. volvacea* strains.

## 4. Discussion

The quality of edible mushroom strains directly affects their production, which is frequently limited by strain degeneration causing huge economic losses (Chen et al., 2017). The selection and breeding of excellent strains is a time-consuming and laborious process, usually involving 1–2 years or even 3–5 years, or more of hard work. However, strain degeneration during succeeding generations reduces strain quality. The low-temperature preservation method can delay the degeneration of fungal strains. For instance, the vitality and ability to produce fruiting bodies of *C. militaris* strain can be maintained for more than 12 months if stored at 4°C (Sun et al., 2018). However, *V. volvacea* cannot tolerate low-temperature preservation, and periodic succession culture is the common method to preserve *V. volvacea* strains which causes strain degeneration after excessive successions (Chen et al., 2019). Sun et al. (2017) found that the successor degenerated strains of *C. militaris* suffer from slow growth rates, defective fruiting body formation and pigment production, low mushroom production, and deformed mushrooms. Kim et al. (2014) reported that the degenerate strain of *Flammulina velutipes* exhibited slow hyphae growth, tight hyphal pads, and little or no fruiting bodies.

The fruiting body of *V. volvacea* is the organized mycelium, which has strong regenerative ability. The generation of tissue cultures requires cutting a small piece of the fruiting body, which is then transferred to a suitable medium for the cultivation of a succession generation (Supplementary Figure S1). In this study, we found that after four successive generations (at T4), the hyphal growth rate, hyphal biomass, and biological efficiency were significantly higher than those of the T0 generation (Figures 1, 3B). However, after many tissue isolation succession, especially after the 12th generation, the *V. volvacea* successor strains showed degenerative symptoms such as decreased hyphal growth rate, prolonged production cycle, and gradual decline in the yield of fruiting bodies. Moreover, for the first time, this study showed that after 18 generations of continuous tissue isolation, *V. volvacea* species completely fails to produce fruiting body (Figure 3A). In addition, we also found an interesting phenomenon; with the increasing generations, *V. volvacea* mycelia branching increased significantly (Figure 2A), and the specific reasons need to be examined in further studies.

Fungal degeneration has been discovered for a long time, but the related causes are poorly understood. Some reports suggested that fungal degeneration could be due to genetic mutations (Zhang et al., 2013), changes in DNA methylation (Xin et al., 2019), invasion of fungal RNA viruses (Magae and Hayashi, 1999), and increased toxins (Yin et al., 2017). Wang et al. (2005) proposed that the degeneration of filamentous fungi may be a symptom of aging involving excessive accumulation of ROS causing oxidative damage to fungal cells (Li et al., 2008). Xiong et al. (2013) experimentally validated that the degeneration of fungal strains was indeed associated with the accumulation of intracellular ROS and overexpression of *gpx* could scavenge intracellular ROS to restore the fruiting ability of *C. militaris* degenerated strain Cm04. This is consistent with our research showing that ROS accumulation increased with the increase in higher generations of *V. volvacea* strain (Figure 4). In particular, ROS amounts increased significantly after 12 successions; the ROS content increased by 50% in the T19 strain compared with the T0 strain, and T19 failed to grow any fruiting bodies.

Organisms have an antioxidant system to avoid excessive ROS damage, which is composed of an enzymatic system and antioxidants, including enzymes such as SOD, CAT, GPX, GR and others (Blagosklonny, 2008). SOD is primarily responsible for removing the active O_2_^−^ species, which is converted to H_2_O_2_ (Gao et al., 2020); CAT decomposes H_2_O_2_ into H_2_O and O_2_ (Ramis et al., 2015), and GPX catalyzes H_2_O_2_ oxidation of glutathione (GSH) to produce oxidized glutathione (GSSG), which is then reduced to GSH (Flohe, 2013). In this study, we found that with the increasing generations, first the activity of SOD decreased, and then the activities of GPX, GR and CAT, in that order, began to gradually decrease. It indicated that SOD is the first line of defense against ROS. The decrease in the activity of antioxidant enzymes in the succeeding strains (Figure 5) aggravated the accumulation of ROS causing serious oxidative damage.

Mitochondria are the main sites of intracellular ROS production, and the excessive oxidative stress from ROS accumulation directly damages the mitochondrial components. This results in more ROS accumulation in a vicious cycle supporting the theory that “mitochondria lead to aging” (Genova et al., 2004; Balaban et al., 2005). The most typical feature of the aging process in yeast is the gradual loss of mitochondrial membrane potential (Jazwinski, 2006), which is consistent with our observations in this study. After several successions, the excessive accumulation of ROS decreased the mitochondrial membrane potential in corresponding *V. volvacea* strains (Figure 7A). Mitochondrial membrane potential reflects the integrity of the mitochondrial structure, thereby affecting the respiratory chain electron transport system, inner and outer membrane integrity, material transport, and mitochondrial shape (Jazwinski, 2006). Mitochondrial fragmentation was noticed in the hyphae of senescent *Podospora anserina* (Scheckhuber et al., 2007), and the loss of mitochondrial function was reported in senescent yeast cells (Borghouts et al., 2004). We also found that mitochondrial morphology changed and bright spots disappeared in the descending degenerate T19 strain (Figure 7B).

The cell nucleus stores the genetic material and regulates cell metabolism, growth, and differentiation (Jiang et al., 2018). Each cell of filamentous fungi contains one or more nuclei. The *V. volvacea* hyphae cells are multinucleated and lack diaphragm-fixed connections; also, the number and size of nuclei are variable in each cell (Chiu and Moore, 1999). Shahi et al. (2015) found clear correlation between the number of *Fusarium oxysporum* mycelium nuclei and the hyphae growth rate; the greater the number of nuclei, the faster the hyphae growth. This is consistent with our results, when the number of nuclei was higher in the early stage of succession, the *V. volvacea* hyphae grew faster (Figures 1B, 2C). Under starvation conditions, in the filamentous fungus *Aspergillus oryzae*, the nuclei of older hyphae are degraded through autophagy to release nutrients for colony survival and growth (Shoji et al., 2010). In *Neurospora crassa*, monocytes lead to cell death due to nuclear senescence after 2–4 transfer cultures (Maheshwari, 2005). In this study, the number of nuclei decreased in *V. volvacea* hyphae with the increasing tissue isolation successions (Figure 2C). This can be attributed to factors such as apoptosis in aging cells, which must be verified in further studies.

**Figure 8:**
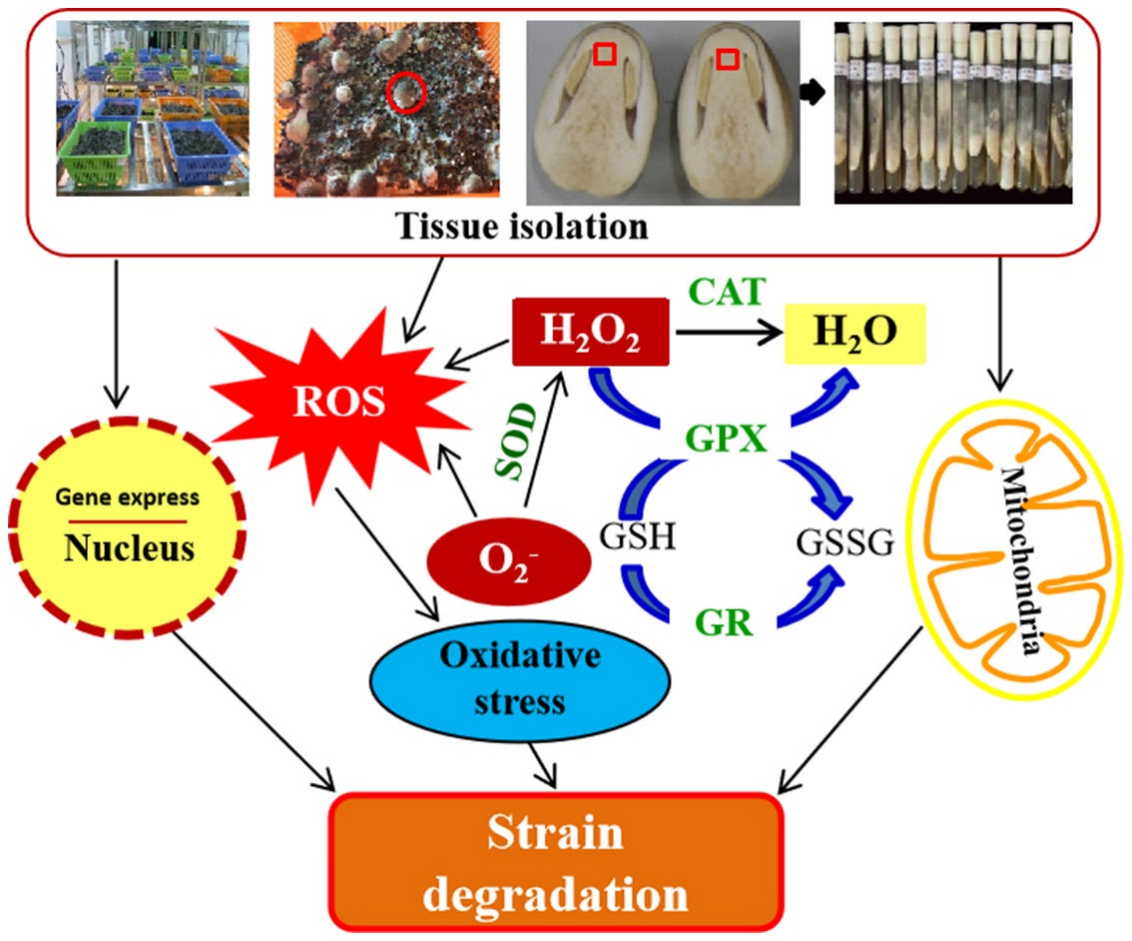
Schematic representation of degeneration mechanism after repeated tissue isolation in *V. volvacea*.

In short, after repeated tissue isolation and subsequent culture in *V. volvacea*, the expression of genes related to ROS scavenging was decreased, the activity of antioxidant enzymes was reduced, and the accumulation of ROS increased, all of these caused damages to the nucleus and mitochondria, resulting in cell aging. In turn, mycelium growth slowed down, mycelial branching and the production cycle increased, production decreased and ultimately failed after the 18th generation (Figure 8). This study proposed for the first time that strain degradation caused by repeated tissue separation of *V. volvacea*, might be due to the strain aging after multiple subgenerations, which resulted in excessive accumulation of ROS and oxidative damage to *V. volvacea*. This study provides a reference for the early identification of species degeneration in *V. volvacea* and a new idea for the control of strain degeneration of *V. volvacea* or other edible fungi by means of biotechnology.

## 5. Conclusion

Tissue isolation is a common method for the rejuvenation and purification of edible mushroom species. This study found that when the tissue of *V.volvacea* species was isolated and passed four times, its production capacity could be effectively improved. However, after 12 successions, degeneration of *V. volvacea* started and the 19th generation completely failed to grow the fruiting bodies. Mechanistically, it happened due to excessive accumulation of ROS which potentially caused cell aging by damaging mitochondria and the nucleus. This study uncovers the details of strain degeneration in *V. volvacea*.

## Data availability statement

The original contributions presented in the study are included in the article/Supplementary material, further inquiries can be directed to the corresponding author.

## Author contributions

JY and FZ designed the research. QW and XA performed the research. QW, QT and, YZ analyzed the data. FZ and QW wrote the manuscript. All authors contributed to the article and approved the submitted version.

## Funding

This work was supported by the National Natural Science Foundation of China (Grant no. 32060708).

## Conflict of interest

XA was employed by Lanzhou Institute of Biological Products Limited Liability Company.

The remaining authors declare that the research was conducted in the absence of any commercial or financial relationships that could be construed as a potential conflict of interest.

## Publisher’s note

All claims expressed in this article are solely those of the authors and do not necessarily represent those of their affiliated organizations, or those of the publisher, the editors and the reviewers. Any product that may be evaluated in this article, or claim that may be made by its manufacturer, is not guaranteed or endorsed by the publisher.

## Footnotes

^1^ http://jgi.doe.gov
