# Supplementary material for: Oxidative damage from repeated tissue isolation for subculturing causes degeneration in Volvariella volvacea
Source: Front Microbiol. 2023 Jul 20;14:1210496. doi: 10.3389/fmicb.2023.1210496 (PMC10397519; doi:10.3389/fmicb.2023.1210496)
Supplement: Supplementary file 1 [file Data_Sheet_1.pdf]

Supplementary Material

1.1 Supplementary Figure

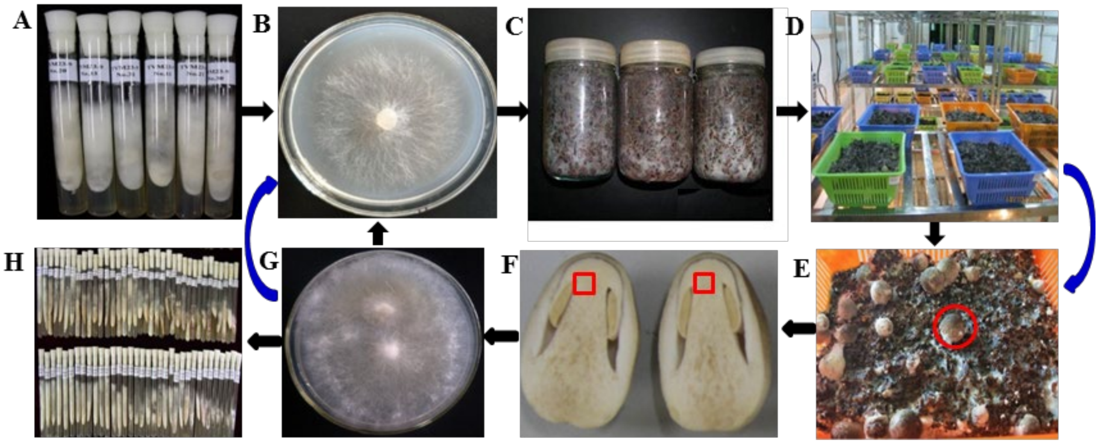

FIGURE S1 The tissue isolation process for succession strains is shown in corresponding images. (A) Original strains (T0), (B) Activation of strains, (C) Seed cultivation, (D) The original base, (E) Egg-shaped period, (F), Tissue separation of the fruiting body, (G) Tissue culture, (H) Strains preservation (T1-T19).

1.2 Supplementary Table

TABLE S1 Sequences of used primer for qRT-PCR.

Gene	Gene symbol	Forward primer	Reverse primer
sod	jgi|Volvo1|118151	CACAAAGACCGCTGCTATC	TAGTAACGACCTCTAGCTTGC
cat	jgi|Volvo1|113089	GCCGCATCGCCATTCTT	GCTTCACCCATACCCAACT
gr	jgi|Volvo1|113291	GCTGTCGTAGGTGCTGGGTA	GGGTCAAATCGCCTCAAA
gpx	jgi|Volvo1|118345	TCGGAGGTGAATGGGAAC	TTGATCCTCGTCAGACCCATA
gapdh	jgi|Volvo1|117975	GCACCAGTGGAAGATGGAATAATG	GGCTTGATGACCACCGTACAT

Note: *sod* = superoxide dismutase; *cat* = catalase; *gr*= glutathione reductase; *gpx*= glutathione peroxidase; *gapdh*= glyceraldehyde phosphate dehydrogenase
